# A 15–20-year follow-up of mental health, psychosocial functioning and quality of life in a single center sample of individuals with differences in sex development

**DOI:** 10.1080/21642850.2022.2116329

**Published:** 2022-09-06

**Authors:** Anne Waehre, Charlotte Heggeli, Kirsten Hald, Anne Grethe Myhre, Trond Diseth

**Affiliations:** a Division of Paediatric and Adolescent Medicine, Department of Child and Adolescent Mental Health in Hospital, Oslo University Hospital, Oslo, Norway; b Centre for Rare Disorders, Oslo University Hospital, Oslo, Norway; c Division of Gynaecology and Obstetrics, Department of Gynaecology, Oslo University Hospital, Oslo, Norway; d Frambu Resource Centre for Rare Disorders, Siggerud, Norway; e Faculty of Medicine, Institute of Clinical Medicine, University of Oslo, Oslo, Norway

**Keywords:** Differences in sex development (DSD), mental health, psychosocial functioning, quality of life, gender identity

## Abstract

**Background:** The aim of the study was to present metal health, psychosocial functioning and quality of life (QoL) of children and adolescents with a difference in sex development (DSD) from their first visit in the newly established multidisciplinary team in 2002–2004 in Norway. A secondary aim was to explore mental health, psychosocial functioning and QoL in the same cohort patient’s as for today and finally explore any childhood predictors for these outcomes in adulthood.

**Methods:** The first part of the study took place in 2002–2004 in a mixed cohort of children and adolescents born with a DSD in 1982–2002, compared to a healthy comparison group. This part involved semi-structured interviews and self-reported and proxy-reported questionnaires. The second part of the study is a longitudinal study of the same participants 15–20 years later (2018–2020).

**Results:** The participants at baseline of the study consisted of 33 patients; 24 assigned females (congenital adrenal hyperplasia, androgen insensitivity syndrome, gonadal dysgenesis and ovotesticular DSD) and nine assigned males; all with a hypospadias diagnosis. Significant differences were found for behavioral and emotional problems between groups, 46, XX females with significant higher total scores on YSR (49.43 + 24.17, *p*  = .047); 46, XY females (21.00 + 12.04, *p*  = .032); and higher internalizing problems scores (YSR) in 46, XX females (16.57 + 9.74), compared with the 46, XY females (5.60 + 5.32, *p* = .047). A positive association between QoL of the participants in adulthood and PedsQL’ social function (*r* = .657, *p* = .020) and psychosocial function in childhood (*r* = .596, *p* = .041) was found.

**Conclusions:** In summary, this study demonstrated that adolescents assigned females with DSD might have more psychiatric problems and a poorer degree of psychosocial functioning compared to a healthy comparison group. As we do find an association with these problems in adolescence and later adult QoL, it is of great importance to respond to these behaviors in early life.

Differences in sex development/disorders of sex development (DSD), formerly described as intersex conditions, are congenital conditions in which development of the chromosomal, gonadal, or anatomic sex is atypical (Diseth, 2008; Lee et al., 2006). DSDs with genital abnormalities occur in approximately one in 4500–5500 live births (Hughes et al., 2007; Thyen et al., 2006). DSD is classified into three subgroups, sex chromosome DSD, 46, XX DSD, and 46, XY DSD.

In 2006, a consensus statement was produced concerning the management of DSD (Lee et al., 2006). A recommendation of this consensus was that evaluation and long-term care for people affected by DSD should be performed at medical centers with multi-disciplinary teams experienced in such conditions. Multidisciplinary DSD teams are supposed to provide psychological support to affected families as a standard component of care, children are informed early about their condition, and gender issues are openly discussed (Hiort et al., 2014). However, historically, health professionals did not openly and fully communicate with patients and families about their DSDs, which today might contribute to the patients with reduced possibility to have an optimal medical and psychological follow-up or frequently being ‘lost to follow-up’ (Lee et al., 2016). The body of literature concerning the psychosocial situation and health-related quality of life of individuals with DSD has increased in recent years, although it remains limited and characterized by contradictory results (Amaral et al., 2015; Roen, 2019).

Previous research has stated that children and adolescents living with DSD born before the consensus statement of 2006 have experienced too little information and inadequate communication about DSD (Lee et al., 2016) with a risk of mental health problems, psychosocial dysfunctioning and poorer QoL later in life. In Norway, psychosocial care for patients and parents were early incorporated in multi-disciplinary teams experienced in DSD around start of 2000. As so, all patients and parents included in this study have received yearly interdisciplinary care included child psychiatric expertise with information and guidance about both current and future medical and psychosocial aspects of the patient`s condition. However, some of the patients were newborns, as others were adolescents with a later presentation for this integrated psychological service, which potentially could affect later quality of life and psychosocial functioning. A follow-up on these young patients QoL and mental health measures later in life is essential.

The aim of the present study was to present mental health, psychosocial functioning and QoL of children and adolescents with a DSD diagnosis from their first visit in the newly established multidisciplinary team in 2002–2004 in Norway, in comparison with healthy same-aged groups. In addition, no long-term follow-up on medical and psychological outcomes have been published in Norway, as so, a secondary aim was to explore mental health, psychosocial functioning and QoL in the same cohort patient’s as for today 15–20 years later and finally explore any childhood predictors for these outcomes in adulthood.

## Methods

### Sample design

**Figure 1.**
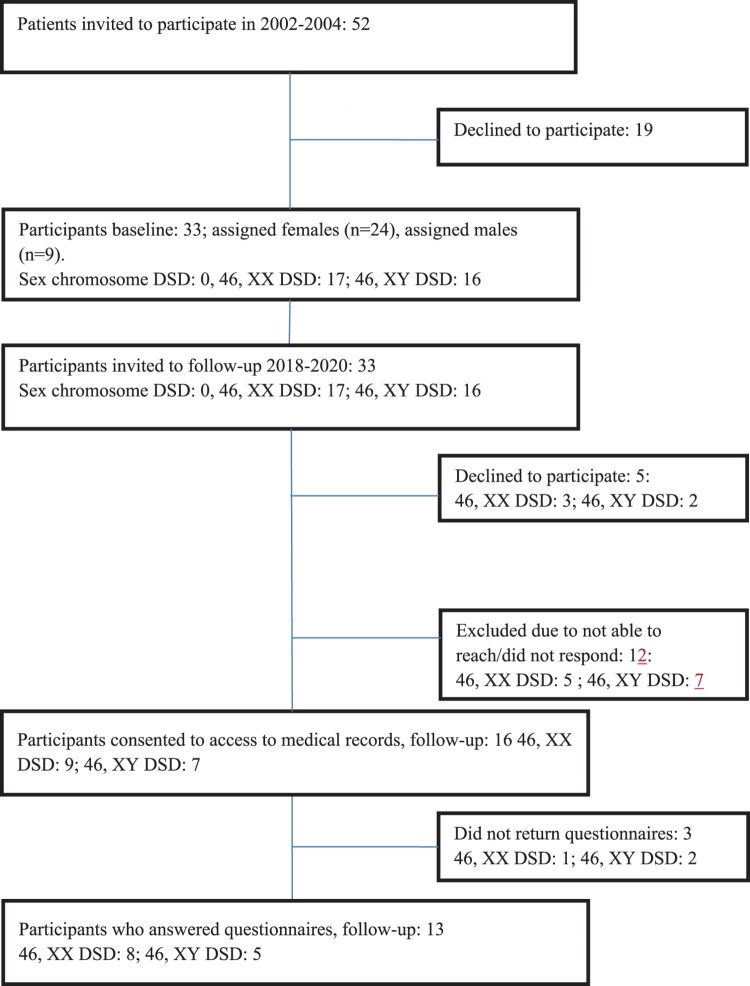
A flow diagram of patients in Part 1 and Part 2.

Initial recruitment took place in 2002–2004 through the Department of Pediatrics at Oslo University Hospital in Norway. Oslo University Hospital is one of two hospitals in Norway that provides multidisciplinary team care for DSD patients. Invited to participate were all DSD patients who visited the newly established multidisciplinary team for children under the age of 18 due to ambiguous genitalia at birth or a later presentation of a DSD diagnosis in the period of inclusion. This initial recruitment were based on the DSD classification of that time and included the diagnosis female and male pseudohermaphroditism, true hermaphroditism and mixed gonadal dysgenesis. Turner and Klinefelter syndrome, patients with syndromes associated with genital malformations and cloacal extrophy were not included. Due to their age span, some of the patients just had their first evaluation for treatment of DSD in the period of inclusion, as others had been diagnosed before and purpose for the visit in the DSD team was a regular follow-up. Altogether 52 patients visited the multidisciplinary team in that time-period and were invited to participate in the study. Thirty-three (63%) accepted the invitation. We reviewed the electronic patients records of these patients (*n* = 33) before the follow-up in 2018–2020 describing DSD phenotypes as defined by the LWPES/ESPE consensus group in 2006 (Houk et al., 2006). A majority were patients assigned females (*n* = 24), a smaller group patients assigned males (*n* = 9). Sixteen (48%) out of 33 individuals consented to participate in the follow-up 2018–2020, however, only 13 returned the questionnaires. Five patients responded that they were not interested in participating, and 12 patients we were not able to reach or they did not respond. See Figure 1 for a complete flow chart of patient selection.

### The control group

The control group for the psychiatric and psychosocial assessments of the adolescents in the baseline part of the study was a sub-sample drawn randomly from a Norwegian epidemiological study of mental health presented in an earlier study (Reinfjell et al., 2006). The study employed stratified, random sampling and two-stage design. In the first stage, screening was carried out by a standardized questionnaire (CBCL). In the second stage, a random sample drawn as a control group from screen-negative (CBCL T-score <60) and screen-positive (CBCL T-score >60) in the same proportion as in the screened general population sample, were invited to participate in a more detailed assessment based on the semi-structured diagnostic interview (CAS and CGAS). Of the 49 adolescents, 35 (71%) and their parents participated. The control group for the preschool children was recruited randomly from a day care center in the south–eastern part of Norway in 2003. Of 33 families invited, 32 children and their parent participated.

### Characteristics of the participants

#### Diagnostic classification

The 33 patients were; based on developmental issues, divided into two age groups; small children, 9 years and younger (born 1993–2001) (*n* = 17), and older children and adolescents, 10–18 years old (born 1984–1992) (*n* = 16). The small children group consisted of females with CAH (*n* = 9) and males with hypospadias (*n* = 8), as the adolescents group were more heterogenetic with a mix of CAH (*n* = 8) and 46, XY females (*n* = 7). However, important to notice is that only one of the patient’s assigned male was in the adolescent group, therefore the statistical analyzes of older children in this study were performed on females only. For diagnostic classifications at baseline and follow-up see Table 1.

**Table 1.** Participants, with diagnosis classification.

		Baseline					Follow-up				
		Total (*n* = 33)	Males^a^ (*n* = 9)		Females^a^ (*n* = 24)		Total (*n* = 13)	Males (*n* = 4)		Females (*n* = 9)	
Age (years)			≤9	10–18	≤9	10–18		≤9	10–18	≤9	10–18
46, XX DSD	CAH (*n*)	17			9	8	8			3	5
46, XY DSD	AIS (*n*)	2			0	2					
	Gonadal dysgenesis (*n*)	4			0	4	1				1
	Ovotesticular (*n*)	1			0	1					
	Hypospadia^b^ (*n*)	9	8	1			4	3	1		
Control group	(Years)		1–6	14–16	1–6	14–16					
	(*n*)	67	15	18	17	17					

CAH: congenital adrenal hyperplasia, AIS: androgen insensitivity syndrome.

^a^ Assigned gender.

^b^ Severe perineoscrotal *hypospadias* associated with genital ambiguity.

#### Surgical characteristics of participants

Thirty-one (94%) of thirty-three participants had a surgery of inner or outer genitalia before first inclusion. Fifteen (88%) of the patients with a diagnosis of CAH (*n* =17) had undergone clitoris reduction; 10 (59%) before the age of 12 months, and five (29%) between the age of 2–9 years. The same patients went through a vaginoplasty at the same time as the clitoris reduction plasty, and one patient had a vaginoplasty only at the age of 4 years. Eight (47%) of the same patients with CAH had two or more genital operations before inclusion due to a new clitoris reduction plasty and/or a vaginoplasty. Four (57%) of the 46, XY females (*n* = 7) had a clitoris reduction and vaginoplasty before the age of 12 months, and all but one had removed the gonads before the age of 4 years. All nine males had undergone a hypospadias operation before the age of 4 years.

Only two of the 16 patients whom consented to access to medical records had new surgery before the follow-up. A third clitoris reduction plasty and vaginoplasty for one patient with CAH, and one patient with 46, XY gonadal dysgenesis had removed the gonads in early 20s.

### Measurements and questionnaires

All participants who agreed to participate in the study at baseline, and their parents, fulfilled validated standardized questionnaires; both self- and proxy reports, and semistructured interviews on their mental health, psychosocial functioning, QoL and socioeconomic status (SES). Furthermore, both patients and parents were interviewed by the child psychiatrist in the DSD team with specific self-constructed questions from the research team covering the individuals and parents view of hospital care management and surgical results. The adults in the follow-up also received a self-constructed questionnaire with questions about SES, sexual functioning, gender identity (male/female/both/other), sexual orientation, experiences with hospital appointments and surgical results. In order to secure patient and public involvement, a reference group was established as part of a larger research project for the follow-up that comprised user participants, LGBTI activists, patient organizations, and professionals with legal, medical, and psychological backgrounds. The reference group represented a variety of gender perspectives and medical- and legal interests.

### Baseline

#### Medical history

Health history and follow-up for a DSD diagnosis were collected from the electronic patient journal (EPJ) by the pediatric endocrinologist or the child surgeon in the DSD team at time of inclusion 2002–2004.

#### Mental health, psychosocial functioning and QoL in childhood

##### Psychiatric assessment

Assessment of the mental health was based on the semi-structured diagnostic interview of patients aged 7–18 years known as the Child Assessment Schedule (CAS) (Hodges et al., 1982) covering aspects of the patient’s life such as school, family, friends, activities, affect-life and behavior. The CAS provides a total problem score; higher score means more mental health problems. Another set of scores consists of symptom complexes scores analogous to psychiatric diagnoses in the DSM.

##### Behavioral and emotional problems and capabilities

For assessing behavioral and emotional problems, and capabilities in children and adolescents, the Child Behavior Checklist (CBCL) and the Youth Self-Report (YSR) were used (Faugli et al., 2009; Javo et al., 2009; Nøvik, 1999). The CBCL were completed by the parents for children aged 15–18 years, and the YSR was completed by children and adolescents between 11 and 18 years old. The CBCL and YSR provide a combined total behavior and emotional score with internalizing and externalizing subscales. Also, eight syndrome scales are calculated, five of these are included in the internalizing and externalizing behavior subscales. The internalizing subscale includes withdrawal, somatic complaints and anxiety/depression. The externalizing subscale includes delinquent and aggressive behavior. Higher scores imply either more symptoms or problems, or better academic or social abilities in other subscales. Raw scores were used in the statistical analyzes.

##### Psychosocial functioning

Overall psychosocial functioning was assessed with Children`s Global Assessment Scale (CGAS) (Schorre & Vandvik, 2004; Shaffer et al., 1983). The score was determined from the total information after the patient and parent interviews. The scores range from 100 (excellent function) to 0 (severe malfunction). A score less than 71 reflect psychosocial problems of clinical significance.

##### Health Related Quality of Life (HRQoL)

HRQoL was measured using the Norwegian version of Pediatric Quality of Life (PedsQl 4.0), (Reinfjell et al., 2006; Varni et al., 1999), consisting of two reports: a child self-report and a parent proxy report that assesses parents’ perceptions of their child’s HRQoL. The two reports are identical and can be grouped into four domains: (1) Physical functioning, (2) Emotional functioning, (3) Social functioning and (4) School functioning. The last three domains make up the Psychosocial Summary Scale, and all subscales are calculated into a Total Summary Functioning score. Higher scores represent a better QoL. The inventory assesses how much of a problem different items have been during the past month, and the results are transformed to a 0-100 scale were higher scores indicate better HRQoL.

#### Parents’ own mental health, psychosocial functioning and QoL

##### Mental health and psychosocial functioning

Occurrence of distress, psychopathology and overall well-being that may have an impact on their child’s mental health and psychosocial functioning, was measured with the General Health Questionnaire (GHQ-30) (Goldberg & Hillier, 1979). The GHQ-30 includes 30 items covering symptoms considered to reflect distress and psychopathology in five dimensions corresponding to anxiety, feelings of incompetence, depression, difficulty in coping, and social dysfunction. The questions are answered on a 4-point scale. The answers to each item have been treated as both Likert scores (0-1-2-3) and ‘case’ score (0-0-1-1) (Malt, 1989). A higher Likert score indicates more psychological distress, and reduced well-being. Clinically important psychopathology is defined as case total scores ≥6.

##### QoL

Overall satisfaction with life in parents was measured with The Quality of Life Scale (QOLS-N) (Wahl et al., 2004) which is a 16-item self-report questionnaire and contains additional information on areas not usually included in HRQoL measures, such as materialistic properties, health, relationships, social activities, education, work, personal development, hobbies, physical activity and independence. The parents are asked to rate their present level of satisfaction with the above-mentioned factors on a seven-point scale. The scale is scored by adding up the items to obtain a total score with possible range 16–112. A higher score depicts a better QoL. Norwegian norms have been established (Wahl et al., 2004).

### Follow-up

#### Medical history adults

Health history and follow-up were collected from the EPJ at time for follow-up in 2018–2020.

#### Mental health, psychosocial functioning and QoL as adults

Occurrence of distress, psychopathology and overall well-being was measured with the General Health Questionnaire (GHQ-30) (Goldberg & Hillier, 1979).

Overall satisfaction with life in the adult DSD patients was measured with The Quality of Life Scale (QOLS-N) (Wahl et al., 2004).

### Statistical analyzes

Continuous variables are presented as the median and range. Categorical variables are presented as quantities and percentages. Data with normal distribution was analyzed using parametric tests and data not normally distributed – with non-parametric tests. The strength of associations between continuous variables was measured using Pearson and Spearman’s correlation coefficients depending on the normality of data distributions. Statistical significance was assumed when *p* < .05. All analyzes were performed using IBM SPSS Statistics 23 and 24.

## Ethics statement

All procedures performed in studies involving human participants were in accordance with the ethical standards of the institutional and/or national research committee and with the 1964 Helsinki Declaration and its later amendments or comparable ethical standards. The Regional Committee for Medical Research Ethics in Norway approved the study (No. 2016/1186).

## Results

### Baseline

#### Socio-demographic variables

There were no significant differences in age or socio-demographic variables between the DSD group and the control group (Supplementary Table 1).

#### Mental health, psychosocial functioning and QoL for the DSD group as a whole

DSD patients total problem score according to the CAS interview are shown in Table 2, indicating more mental health problems in the DSD group than in the control group. Also, on the anxious/depressed subscale on the CBCL questionnaire the DSD group scored significally more symptoms than the control group. Furthermore, the DSD group as a whole, including small children to adolescents, had significantly lower overall psychosocial function (CGAS) scores than the control group (Table 2).

**Table 2.** DSD group compared with the control group.

	DSD group (*n *= 32)	Control group (*n *= 67)
Gender, *n* (%)		
Female	24 (80.0)	34 (50.7)
Male	9 (20.0)	33 (49.3)
Age at study (years)	9.5 (2–18)	14 (1–16)
CAS^a^	*n *= 20	*n = *35
Total score	28 (4–72)^a^	16 (3–64)^a^
CGAS^b^	*n = 32*	*n = 35*
	77.50 (40–94)^a^	85 (55–98)^a^
YSR, T-scores	*n = *14	*n = *34
Total score	34 (4–81)	22.5 (0–86)
Internalizing	11.5 (0–32)	5.5 (0–30)
Externalizing	10 (2–31)	7.5 (0–45)
CBCL	*n *= 27	*n *= 61
Total score	15 (3–74)	11 (0–76)
Internalizing	4 (0–19)	4 (0–19)
Externalizing	5 (0–28)	4 (0–39)
CBCL Subscales		
1 Withdrawn	1 (0–7)	1 (0–2)
2 Somatic complaints	1 (0–8)	1 (0–5)
3 Anxious/depressed^a^	2 (0–10)^a^	1 (0–5)^a^
4 Social problems	1 (0–10)	0 (0–5)
5Thought problems	0 (0–2)	0 (0–0)
6 Attention problems	2 (0–10)	1 (0–7)
7 Delinquent behaviour	1 (0–5)	0 (0–4)
8 Aggressive behaviour	3 (0–24)	4 (0–19)
PedsQl – Parent report	*n = *26	*n = *27
Total score	78.3 (53.3–98.9)	84.7 (56.5–100.0)
Physical health	82.8 (43.8–100.0)	87.5 (59.3–100.0)
Emotional function	72.5 (35.0–100.0)	80.0 (55.0–100.0)
Social function	85.0 (45.0–100.0)	90.0 (65.0–100.0)
School function	80.0 (0.0–100.0)	80.0 (0.0–100.0)
Psychosocial function	80.0 (48.3 –98.3)	83.3 (45.0–100.0)

^a^ Difference between groups is significant at *p* < .05. Median (range) if not stated otherwise.

^b^ CGAS-scores from child interviews when given, or else from parent interviews.

#### Age group 1–9 years

Differences in children in age groups 1–9 years old were investigated using the CAS, CGAS, CBCL and PedsQL both children and parent reported validated questionnaires. No significant differences were found between controls, neither different genders nor diagnoses on any scales or measures in this sample.

#### Age group 10–18 years: assigned females with DSD compared with control group, and 46, XX females vs. 46, XY females (Table 3 and 4)

CGAS total score, the total raw scores of CBCL and YSR, externalizing, and internalizing scores and CAS total score are listed in Tables 3 and 4 of both 46, XX and 46, XY females and the female control group. The scores for 46, XX females and the 46, XY females were statistically significantly lower in overall psychosocial function (CGAS) than the control group. Interestingly, significant differences were found for behavioral and emotional problems between groups, where 46, XX females had a significant higher total score on YSR than 46, XY females. The 46, XX females also had significantly higher scores on both internalizing problems (YSR) and different subscales of the YSR compared with XY females; both withdrawn behavior, social problems and attention problems.

**Table 3.** Adolescents assigned 46, XX females, 46, XY females and control group (age 10–18 years).

Median (min–max)	46, XX females (*n *= 8)	46, XY females (*n *= 7)	Females control (*n *= 17)
Age at study (years)	14 (10–18)	14 (10–18)	15 (14–16)
CGAS^a^	*n *= 8	*n *= 7	*n *= 17
Total score*	68 (54–85)*	65 (40–90)*	85 (65–98)*
*n* (%); < 0–71	4 (50.0%)	5 (71.4%)	1 (5.9%)
YSR raw scores	*n = 7*	*n = 5*	*n = 17*
Total score*	50 (19–81)*	21 (4–38)*	24 (0–61)
Internalizing*	16 (3–32)*	4 (0–14)*	6 (0–30)
Externalizing	9 (4–31)	10 (2–11)	7 (0–24)
CAS	*n *= 7	*n *= 7	*n *= 17
Total score	32 (15–67)	13 (4–72)	13 (3–64)
CBCL raw scores	*n *= 7	*n *= 6	*n *= 17
Total score	12 (4–74)	14 (3–39)	10 (0–46)
Internalizing	5 (2–19)	4.50 (2–12)	4 (0–19)
Externalizing	3 (0–28)	6 (0–18)	3 (0–13)

Median (min–max).

* = *p*  < .05.

^a^ Either based on child interviews, or else parent interviews.

**Table 4.** Adolescents, 46, XX DSD vs. 46, XY DSD females, without control (age 10–18 years).

Median (min–max)	46, XX females (*n *= 8)	46, XY females (*n *= 7)
YSR subscales	*n *= 7	*n *= 5
Withdrawn*	4 (2–6)	2 (0–3)
Somatic complaints	4 (0–13)	1 (0–2)
Anxious/depressed	8 (0–14)	2 (0–9)
Social problems*	3 (0–6)	1 (0–2)
Thought problems	2 (1–10)	1 (0–5)
Attention problems*	7 (4–11)	1 (0–4)
Delinquent behavior	3 (0–9)	3 (1–4)
Aggressive behaviour	6 (4–22)	7 (1–8)
PedsQl – Child report	*n = 6*	*n = 7*
Total score	73.91 (41.30–92.39)	89.13 (71.74–95.65)
Physical health	90.62 (37.50–100)	96.87 (87.50–100)
Emotional function	55 (50–100)	80.0 (55–100)
Social function	90 (25–100)	100.0 (60–100)
School function	60 (55–75)	82.50 (55–90)
Psychosocial function	71.67 (43.33–88.33)	89.17 (56.67–93.33)
PedsQl – Parent report	*n = 7*	*n = 6*
Total score	85.87 (57.61–86.96)	80.43 (53.26–97.83)
Physical health	81.25 (65.62–96.87)	90.63 (43.75–100)
Emotional function	75 (55–80)	72.5 (35–90)
Social function	85 (45–100)	77.5 (65–100)
School function	80 (45–100)	72.50 (35–100)
Psychosocial function	80 (48.33–91.67)	74.17 (53.33–96.67)

Median (min–max).

*Difference between groups is significant at *p* < 0.05.

In the older children/adolescent group the self-assessment HRQL using PedsQL, showed a clear tendency of lower score in the female group with 46, XX compared to the females with 46, XY, however not significant. The physical health dimension had the highest score. The lowest scores were in emotional and school function.

#### Parent-reported medical details and own experiences from clinical concerns and care at baseline; 2002–2004

Only about half of the parents were happy with the results of surgery on outer genitalia of their children, both parents of assigned females and males. When the parents were asked if it was difficult for the child to talk about the diagnosis, 34.7% of the assigned females and only 11.1% of the assigned males were in such situation.

### Follow-up

#### Mental health

About half (7/13) of the participants in the follow-up reported clinically important psychological distress or mental health problems as defined as case total scores ≥6 on GHQ 30. See Table 5 and Supplementary Table 3 for details.

**Table 5.** Quality of Life (QOLS), distress, psychopathology and well-being (GHQ-30), follow-up. (*N* = 13).

	Median (min–max)
Age first inclusion	10 (5–18)
Age follow-up	26 (21–34)
QOLS	
Total score	89 (49–111)
GHQ 30	
Lickert score	29 (18–68)
Case score	7 (0–26)
Case, 6<^a^	7 (53.8%)^1^

^a^ For GHQ 30, clinically important psychopathology is defined as case total scores ≥6.

#### QoL

The scores of the QOLS by the 13 participants were equally distributed above and below the Norwegian average, and the mean of the participants (83.46 + 19.13) was not significantly different from the Norwegian population (84.10 + 12.5) (Wahl et al., 2004), however, the variation among patients was large between a score of 49–111. See Supplementary Table 3 for details. Two of the patients from the initial small children group, and four from the adolescent group had QOLS total score below the QOLS norms or reference values from the general Norwegian population. No difference in QOLS total score were found between the males and females in the current sample.

#### Patients-reported health-related data and the patient’s own experiences as adults

Self-reports from the 13 patients that completed the questionnaires are listed in Supplementary Table 2 and shows a great variation in satisfaction with external genitalia in the group. Ten of 12 participants had mainly positive experiences with post-surgery follow-ups, but only 7/11 had mainly positive experiences with the information given before surgery. For more details see Supplementary Table 2.

#### Sexual orientation and gender identity as adults:

##### Sexual orientation as adults

Less than half of the nine (4/9) female participants in the follow-up reported a heterosexual orientation, as did three out of four of the assigned males.

##### Gender identity as adult

When asked as adults, all nine female participants reported that their identity today corresponds with assigned gender. One of the female patients reported that she had a feeling that her gender identity in childhood did not correspond with assigned gender. One of four male participants reported a gender identity that did not correspond with assigned gender.

##### Gender reassignment

Of the 16 patients that consented to participate in the follow-up, two patients had performed gender reassignment. One patient with a CAH diagnosis of the salt-wasting form of 21-hydroxylase deficiency, assigned female gender and operated with clitoris reduction plastic and vaginoplasty at the age of 6 months, is today living as a male. In the medical records it is reported that the patient had poor compliance concerning hydrocortisone treatment and in early childhood had a feeling of being a boy. The patient started testosterone treatment at the age of 16 years and performed mastectomy at the age of 18 years. Another patient diagnosed with 46, XY gonadal dysgenesis and assigned female at birth, had performed gender reassignment to male. The patient stopped estrogen treatment at the age of 17 years after changing social sex at 16 and at 19 years old started testosterone treatment and in his 20s performed a modified metaidoioplasty.

#### Childhood predictors for QoL in adulthood

Positive associations between QoL of the participants in adulthood and PedsQL’ social function (*r* = .657, *p* = .020) and psychosocial function in childhood (*r* = .596, *p* = .041) were found.

#### Associations between adults’ mental health, QoL and other variables such as satisfaction with genitalia

All 6 patients with a QOLS total score below the normative data did have a score defined as «cases» at the GHQ 30. Higher QoL (QOLS) correlated negatively with lower psychological distress (GHQ-30) (*r* = −.88, *p* = .000). There were positive associations between QoL and satisfaction with outer genitalia (*r* = .77, *p* = .004). Negative associations were found between psychological distress (GHQ-30) and satisfaction with outer genitalia (*r* = −.85, *p* = .000).

#### Mental health, QoL and patient-reported health care management

Significantly lower psychological distress (GHQ-30) were discovered in the group who had mainly positive experiences with the information given before surgery (25.00 ± 8.06), than the ones with mainly negative experiences (58.00 ± 7.48). Also, higher QoL were found in the group who had mainly positive experiences with the information given before surgery (97.00 ± 9.71), compared to the ones with mainly negative experiences (60.25 ± 10.05).

## Discussion

Our main results suggest more psychopathology in the adolescents with a DSD diagnosis compared to controls in general, and females with 46, XX karyotype with more psychosocial problems compared to females with 46, XY in particular. Interestingly, an important finding is an association between self-reported social and psychosocial function in childhood with later adult QoL. However, caution has to be drawn as this study was hampered by a low sample size in general, and a smaller and heterogeneous follow-up.

When looking at the total cohort of children and adolescents with a DSD diagnosis at baseline in 2002–2004, the present study indicated more psychiatric problems; most of the problems were internalizing in nature due to higher levels of anxiousness and depression, together with a lower psychosocial functioning compared to a healthy comparison group. However, we found no increased risk of psychological problems, psychosocial problems or poor QoL in younger children (1–9 years old) with DSD compared to controls, both in the self-reports and the parent-reports. There are studies supporting such findings (Jürgensen et al., 2007), while others report the opposite (Slijper et al., 1998). One study found that age was negatively associated with QoL, indicating that there is a deteriorating effect as the child gets older (Mahesh, 2019). One possible explanation is that people with DSD might struggle more with age, getting increasingly aware of their condition, especially when seeking out relationships (Johannsen et al., 2006).

The present study found lower self-reported overall psychosocial functioning in teenage females with DSD compared to the female control group at baseline in 2002–2004. In particular, the 46, XX assigned females in this cohort, exclusively patients with a CAH diagnosis, self-reported more emotional and behavioral problems than the 46, XY assigned females. Specifically, internalizing problems with withdrawn behavior and social problems, as well as attention problems were reported. Even though these key findings are from two decades ago, more recent studies support these findings (Ediati et al., 2015; Kung et al., 2018). In line with the results in this study teenage girls with CAH have been found to more likely isolate themselves socially (Ediati et al., 2015), which, together with stigmatization are discussed to increase psychological distress (Kuhnle & Krahl, 2002; Warne & Raza, 2008). Given these risks, young individuals with a CAH diagnosis should be given psychological and social support. Interestingly, the 46, XY females showed better psychological health than both the 46, XX females and the control group, although not significantly so. However, comparing 46, XX females and 46, XY, females might not be of much relevance. Some of the 46, XY females got their diagnosis late in adulthood with less hospital contact from early age, different follow-up and with or without androgenization prenatal.

When it comes to self-assessment of HRQOL in the teenage group, at baseline in 2002–2004, there was a clear tendency of lower score in the assigned female group with 46, XX compared to the assigned females with 46, XY. The lowest scores were in emotional and school function. The reduced school functioning in adolescents 46, XX females, might be in due to increased risk of cognitive impairment. Children and adolescents with CAH are at risk of adrenal crisis with salt loss and hypoglycemia that may negatively impact cognitive performance (Hamed et al., 2018). As so, some studies have highlighted the need to assess cognitive functions in children and adolescents with CAH (Amr et al., 2019; Hamed et al., 2018). However, a newly published study of children and adolescents diagnosed through the neonatal screening program for CAH in Sweden showed normal cognitive abilities (Valeria Messina et al., 2020). Nevertheless, early intervention in children with cognitive deficits is crucial to prevent learning deficits later in childhood, and especially when combined with attention problems, which also was found in this cohort.

About half of the participants, when assessed as adults, scored high on self-reported psychological distress. Also, other studies have found psychological problems to be apparent in adults with different DSD diagnoses, and inferior to children (Kleinemeier et al., 2010). However, other studies do not find any psychological or psychosocial struggles (Migeon et al., 2002; Stikkelbroeck et al., 2003; Warne et al., 2005). Two quite recent studies in Sweden employed nation-wide population-based registers of CAH and psychiatric diagnoses to investigate if mental health outcomes differed between individuals with CAH and age- and sex-matched controls (Engberg et al., 2015). Elevated rates of alcohol misuse, substance use disorders, and stress-related and adjustment disorders were found in females with CAH (Engberg et al., 2015). However, other studies find no psychiatric comorbidities in CAH (Kung et al., 2018; Reisch et al., 2011).

The QoL measures from the current cohort are in line with Norwegian norms (Wahl et al., 2004). However, the scores were scattered between 49 and 111, with half of the patients having scores below the norm, and half above. These mixed results may warrant clinicians that within the DSD population, some individuals may show resilience against developing difficulties and other might have more challenges. Studies have earlier suggested a lot of different possible risk factors for reduced QoL and increased psychological distress among the DSD patient group: medical and surgical decision-making, informing others about the condition, appearance differences, compromised fertility and sexual functioning and issues related to psychosexual identity (Johannsen et al., 2006; Kuhnle et al., 1995; Nordenskjold et al., 2008; Warne et al., 2005). Other studies have pointed at sex reassignment procedures and genital surgery (Slijper et al., 1998).

In the current cohort, however small, there was no association between the number of surgeries or hospital admissions with QoL or psychological distress in adulthood. Nevertheless, there was found an association between satisfactions with the information given before surgery with good QoL and less psychological distress in adulthood. Furthermore, although the consensus today is to recommend early disclosure to patients, earlier days it was not uncommon that medical doctors did not inform the patients about previous surgeries. Good information about the diagnosis has been found to improve coping skills and adjustment to diagnosis (Cohen-Kettenis, 2010). Psychological support, both social and professional, has been positively connected to symptoms of QoL and psychological distress in both childhood and adulthood (Kanhere et al., 2015; Schweizer et al., 2017). One study found a connection between general psychopathology occurring twice as often in children not having psychological counseling at the diagnosis time (Slijper et al., 1998). Another study found that patients with CAH with regular follow-up after going into adult care had better psychological health and QoL than the group who did not have regular follow-up (Bachelot et al., 2015). Although Norway early integrated psychological services into their team, some of the participants in this study were older adolescents at baseline who might not have had proper age-adjusted information growing up. Unfortunately, due to the small follow-up no differences can be drawn between those born before and after introduction of multidisciplinary teams with psychological support. Indeed, still many parents find it difficult to divulge the diagnosis to their children.

Interestingly, less than half of the nine female participants in the follow-up reported a heterosexual orientation. This is in line with a quite recent published review that indicates that females with CAH had a greater likelihood to not have an exclusively heterosexual orientation than females from the general population (Daae et al., 2020). As so, clinicians also need to be open for a variation in non-heterosexual orientation in young and adults with CAH and address sexual orientation when indicated and if relevant. The dsd-LIFE study reported a gender variance in 4% of the 1040 participants (Kreukels et al., 2018). In this study, when asked as adults, all nine female participants reported that their identity today corresponds with assigned gender. One of the female patients reported that she had a feeling that her gender identity in childhood did not correspond with assigned gender, and one of four male participants reported a gender identity that did not correspond with assigned gender. Besides, two of the 16 patients that consented to participate in the follow-up had performed gender reassignment. Importantly, as this study also shows, clinicians should be aware of the gender identity variance and be open-minded to the increasing knowledge of non-binary identity in general.

### Limitations

Overall, this study was hampered by a low sample size in general, which was expected because of the rarity of the conditions, combined with response numbers. Because of missing participants in the male teenager group in part 1 of the study; partly due to the gender assignment practice at that time, it was not possible to compare across genders. Another limitation is the small sample in the follow up study, which might bias any statistical analyzes, and made it difficult to analyze across groups of diagnoses or assigned genders. Many refused to take part in the follow-up study, which could mean that the mental health and QoL of patients are further compromised, since in general the no responders tend to have poorer health, may be depressed or socially isolated (Zainuddin et al., 2013).

## Conclusion

In summary, although a small sample size, and smaller follow-up, this study demonstrated that adolescents assigned females with DSD might have more psychiatric problems and a poorer degree of psychosocial functioning compared to a healthy comparison group. Importantly, the female adolescents with a CAH diagnosis in this local cohort do report more internalizing emotional and behavioral problems, including social problems, withdrawn behavior, and attention problems. As we do find an association with these problems in adolescence and later adult QoL, it is of great importance to respond to these behaviors in early life. The study demonstrates that emotional and behavioral adjustment in adolescents with CAH may be particularly important because it sheds light on how early any CAH-related adjustment differences emerge. Another important finding was that higher satisfaction with information before surgery is associated with better QoL and lower psychological distress. This highlights the importance of having a psychological aspect in the follow up of children and adolescent patients, and also make sure that the information before surgery are understood by the patients, and not only by their parents.

## Author’s contribution

A.W. and C.H. wrote the main manuscript text and C.H. prepared figures and tables. T.D. included patients in the first part of the study, and A.M. and K.H. included patients in the second part of the study. All authors reviewed the manuscript.

## Informed consent

We obtained informed consent from all individual participants included in Part 1 and Part 2.

## Data Availability

The data that support the findings of this study are available on request from the corresponding author.
